# Surgical management of a superficial femoral artery aneurysm in a patient with a high risk of thromboembolism

**DOI:** 10.1093/jscr/rjaf490

**Published:** 2025-07-11

**Authors:** Kazuki Mori, Masazumi Kume, Ryotaro Nagashima, Ken Nakayama, Eisuke Kawakubo

**Affiliations:** Department of Vascular Surgery, NHO Beppu Medical Center, 1473, Uchikamado, Beppu, Oita 874-0011, Japan; Department of Vascular Surgery, NHO Beppu Medical Center, 1473, Uchikamado, Beppu, Oita 874-0011, Japan; Department of Vascular Surgery, NHO Beppu Medical Center, 1473, Uchikamado, Beppu, Oita 874-0011, Japan; Department of Vascular Surgery, NHO Beppu Medical Center, 1473, Uchikamado, Beppu, Oita 874-0011, Japan; Department of Vascular Surgery, NHO Beppu Medical Center, 1473, Uchikamado, Beppu, Oita 874-0011, Japan

**Keywords:** thrombus, superficial femoral artery, femoral artery aneurysm, antiphospholipid syndrome, vasculitis

## Abstract

A 62-year-old man with a history of proteinase 3-specific antineutrophil cytoplasmic antibody-associated vasculitis, antiphospholipid syndrome, and deep vein thrombosis presented with an asymptomatic 15-mm-diameter right superficial femoral artery (SFA) aneurysm, showing increased intraluminal thrombus on computed tomography compared to 2 years prior. Despite anticoagulation, thrombus progression prompted surgery to prevent embolization. The right SFA atherosclerotic aneurysm was resected. A reversed femoral vein graft was used for arterial reconstruction. Three-year follow-up computed tomography showed no aneurysm or pseudoaneurysm at the reconstructed site. Surgery was successful in this case of a right SFA aneurysm at a high thrombosis risk.

## Introduction

Superficial femoral artery (SFA) aneurysms are a rare subtype of peripheral arterial aneurysms [1, 2]. SFA aneurysms are often caused by infections, trauma, or iatrogenic factors, with an atherosclerotic etiology being relatively rare. Owing to the potential for rupture and thromboembolic ischemic complications, early surgical intervention is necessary. Here, we report the surgical resection of an atherosclerotic SFA aneurysm in a patient at high risk of thrombosis.

## Case report

**Figure 1:**
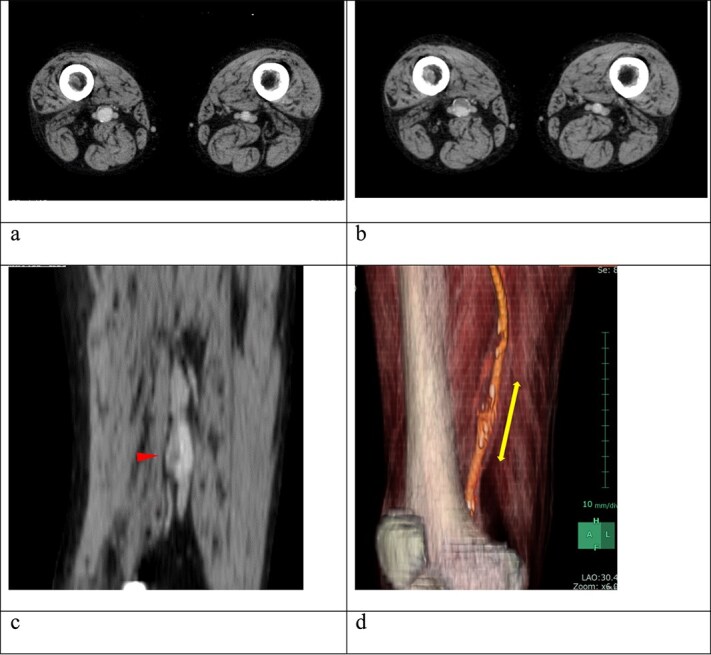
(a) Contrast-enhanced computed tomography (CT) performed 2 years earlier showed no thrombus in the right superficial femoral artery (SFA) aneurysm. (b) Despite ongoing oral anticoagulation therapy, preoperative contrast-enhanced CT revealed thrombus progression within the aneurysm, which was located 6 cm proximal to the knee and measured 15 mm in diameter. (c) The sagittal section of the contrast-enhanced CT showed an irregular thrombus on the inner wall of the aneurysm (arrow). (d) A three-dimensional-reconstructed contrast-enhanced CT identified the SFA aneurysm (arrow).

A 62-year-old male patient presented with an asymptomatic 15-mm-diameter right SFA aneurysm on computed tomography (CT). The patient had a history of chronic limb ischemia of the left upper extremity due to proteinase 3-specific antineutrophil cytoplasmic antibody (PR3-ANCA)-associated vasculitis. He also had a history of deep vein thrombosis (DVT) and pulmonary embolism secondary to antiphospholipid syndrome (APS) and systemic lupus erythematous, requiring pulmonary thromboembolectomy, right deep vein thrombectomy, and inferior vena cava filter placement. Although the 12-mm-diameter SFA aneurysm was detected at the time of DVT evaluation, no further imaging follow-up was performed after DVT treatment. Given the aforementioned medical history, the patient was on anticoagulation therapy with 10 mg/day of rivaroxaban and immunosuppressive therapy with 6 mg/day of methylprednisolone and 100 mg/day of azathioprine. A contrast-enhanced CT revealed the right SFA aneurysm, measuring 15 mm in diameter. The aneurysm was located ~6 cm proximal to the knee joint. A comparison with the CT scan for DVT recurrence evaluation obtained 2 years prior revealed an increase in the intraluminal thrombus within the aneurysm (Fig. 1). CT angiography of the distal lower extremity revealed no evidence of occlusion and recurrent DVT.

Despite the implementation of anticoagulation therapy, the intraluminal thrombus within the aneurysm continued to grow. Consequently, surgical resection was deemed necessary to prevent potential embolization.

The SFA was dissected through a medial femoral incision. The aneurysm was carefully dissected from the surrounding tissues, which were densely adherent. Subsequently, the femoral vein (FV) was dissected, and a 3-cm segment was harvested as a graft. Following intravenous heparinization, the SFA was occluded, and the aneurysm was resected. The aneurysm was filled with a mix of red and white thrombi, and the aneurysm wall exhibited atherosclerotic changes. A reversed FV graft was interposed between the proximal and distal segments of the artery using 6-0 polypropylene sutures for end-to-end anastomosis.

**Figure 2:**
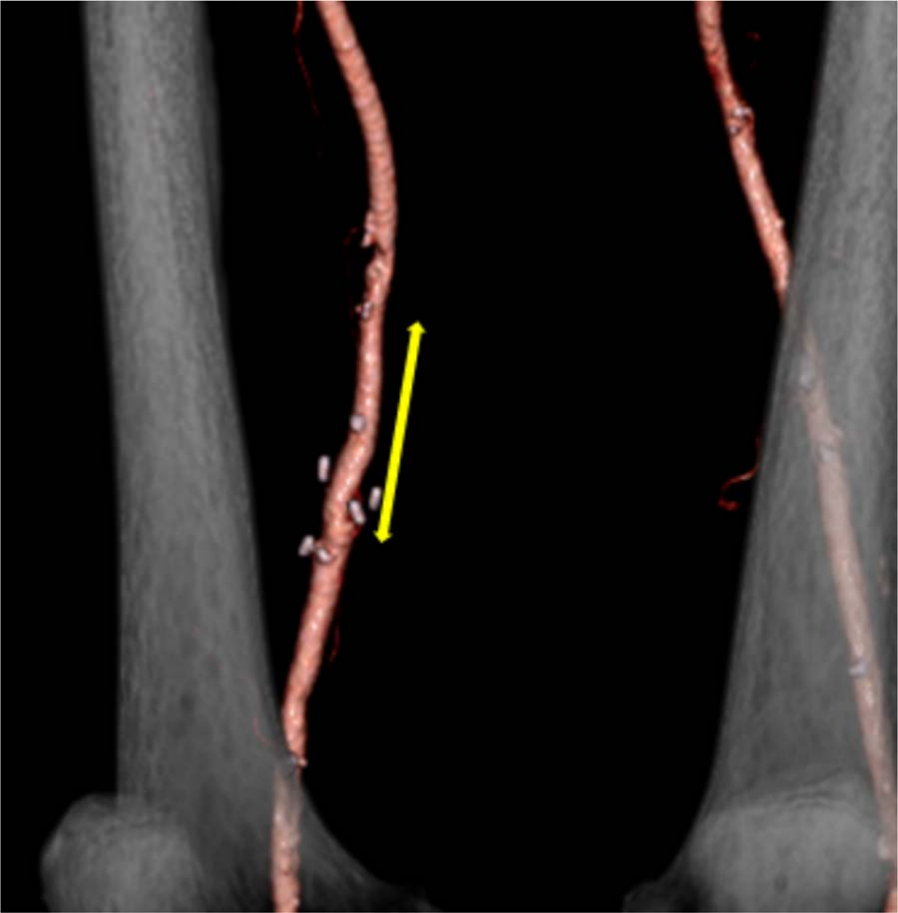
Postoperative contrast-enhanced CT revealed no aneurysm or stenosis formation at the reconstructed site (arrow) and optimal visualization of distal vessels.

On pathological examination, the resected aneurysm wall revealed no findings other than atherosclerotic changes. Postoperative lower extremity edema was not observed despite using the FV as a graft. Postoperative CT showed no evidence of complications at the reconstructed vascular site (Fig. 2). Postoperatively, the patient was initiated on warfarin therapy. No aneurysm or pseudoaneurysm formation was observed at the reconstructed site on follow-up CT 3 years postoperatively.

## Discussion

Peripheral arterial aneurysms frequently involve the popliteal and common femoral arteries. Stenotic lesions greatly occur in the SFA, making SFA aneurysms rare [1, 2]. Posttraumatic pseudoaneurysms and those associated with infections or connective tissue disorders are the most common causes of SFA aneurysms. They are frequently associated with aneurysms in other locations and detected during aortic aneurysms investigations [3, 4]. Bilateral occurrence is observed in 13% of the cases [5].

The SFA is located deep within the thigh, making it difficult to palpate aneurysms until they become quite large, often leading to an asymptomatic course [6]. SFA aneurysms have been observed to present with rupture as the initial symptom [2, 4]. In addition, as with other peripheral aneurysms, SFA aneurysms are at risk of thrombosis and embolization, which can lead to limb-threatening ischemia [2, 3]. The lower incidence of initial ischemic events in SFA aneurysms compared with popliteal artery aneurysms is hypothesized to be attributable to their location outside the joint [6].

Although no consensus has been reached regarding the optimal surgical management of SFA aneurysms, surgical intervention is generally indicated in symptomatic patients, such as those with rupture or ischemia [1, 2]. Surgical intervention should be considered for asymptomatic aneurysms with a diameter > 25 mm or a saccular morphology [1, 3, 6]. Although the patient was asymptomatic and the aneurysm was initially a small, 15-mm fusiform lesion, the intraluminal thrombus increased over 2 years despite anticoagulation therapy. There was a concern for further thrombus formation because of underlying APS. In addition, the patient had ANCA-associated vasculitis, which was considered to increase the risk of severe ischemia in arterial occlusion.

Treatment options for SFA aneurysms include aneurysm resection with graft replacement or bypass surgery [1, 3, 5]. Simple ligation may be considered when the sufficient collateral flow from the profunda femoris artery is present, and reconstruction is challenging [7]. Resections of the aneurysm and graft replacement are generally considered the first-line treatment [1, 6]. Because of the straight configuration of the SFA segment, prosthetic grafts can be considered an alternative to autogenous vein grafts. Because of the patient’s immunosuppressed state, an autogenous vein graft was used to reduce the risk of postoperative graft infection. The great saphenous vein is frequently utilized as the autogenous vein graft. However, due to the patient’s history of APS and DVT, this patient had a high risk of recurrent DVT because the great saphenous vein is an important collateral vessel. The FV was used as the graft to mitigate the risk of impaired venous return in the event of recurrent DVT. The FV was free of thrombus on the preoperative image, with an appropriate diameter, making it an ideal graft. FV grafts have been shown to be effective autologous vein grafts, provided that a preoperative lower limb vascular evaluation is conducted [8]. The graft exhibited excellent outcomes, with no indications of aneurysm formation, even after 3 years.

Several studies have reported endovascular treatment with stent grafts [9–11]. Endovascular therapy is widely regarded as the preferred treatment option for older or high-risk patients. In this case, the primary objective was to prevent thromboembolism. Endovascular treatment was not chosen because of concerns about the potential for embolization caused by catheter manipulation within the vessels.
